# Investigation of CeO_2_ nanoparticles on the performance enhancement of InsulatingOils

**DOI:** 10.1016/j.heliyon.2023.e19264

**Published:** 2023-08-21

**Authors:** Obaidur Rahman, Arif Ali, Ahmad Hussain, Shakeb Ahmad Khan, Mohd Tariq, Shabana Urooj, Lucian Mihet-Popa, Qasim khan

**Affiliations:** a Department of Electrical Engineering, ZHCET, Aligarh Muslim University, Aligarh, 202002, India; b Department of Mechanical Engineering, Indian Institute of Technology, Ropar, 14001, India; c Department of Electrical Engineering, Jamia Millia Islamia, New Delhi, India; d Department of Electrical Engineering, College of Engineering, Princess Nourah Bint Abdulrahman University, P.O. Box 84428, Riyadh, 11671, Saudi Arabia; e Faculty of Information Technology, Engineering and Economics, Oestfold University College, 1757, Halden, Norway; f Texas A&M University, College Station, TX, 77840, USA

**Keywords:** Insulating oil, Nanodielectric fluid, Breakdown strength, Dielectric constant, Nanoparticle, Aging, Dissipation factor

## Abstract

Integrating nanotechnology in dielectric fluid significantly inhibits losses and boosts overall dielectric fluid performance. There has been research done on the effects of introducing various nanoparticles, such as titania, alumina, silica nanodiamonds, etc. In this paper, a novel nanoparticle, Ceria (CeO_2_), has been used, and its properties were examined using the FTIR (Fourier Transform Infrared) spectrum, the XRD (X-ray Diffraction) spectrum, the SEM (Scanning Electron Microscopy), and the TEM (Transmission Electron Microscopy). This paper illustrates an efficient dielectric fluid prepared by the successful dispersion of Cerium Oxide (CeO_2_) nanoparticles in various concentrations into four commercial oils, namely mineral oil, rapeseed oil, synthetic ester oil, and soybean oil, to enhance and improve their dielectric characteristics. The performance investigation emphasises breakdown strength enhancement and other dielectric properties of the colloidal solution comprising different nanoparticle (NP) concentrations. Various commercial oils are used as a base in nano-oil to diversify their applicability as dielectric fluids by measuring the correlation in dielectric parameters and statistically assessing their applicability with normal and Weibull distributions. The obtained experimental data sets were analyzed using the Statistics and Machine Learning Toolbox in MATLAB. The aging measurement has been done only on mineral oil, and results were matched using a predictive model of statistics and the Machine Learning Toolbox in MATLAB. Well-dispersed CeO_2_ NPs in the insulating oils lead to a significant increase in AC breakdown strength. The effect of ageing on the dielectric properties of nano oils yields better results than conventionally aged oil. It has been observed that the breakdown voltage is enhanced by up to 30% for mineral oil at an optimal concentration of 0.01 g/L, 9% for synthetic ester oil at 0.03 g/L, 18% for rapeseed oil at 0.02 g/L, and 19% for soybean oil at 0.03 g/L nanoparticle concentration. Following the dispersion of CeO_2_ nanoparticles, the dielectric constant of all insulating oils has also significantly improved.

The overall experimental results are promising and show the potential of the CeO_2_ NPs-based nano oil as an efficient and highly performing dielectric oil for different power applications.

## 1 Introduction

Dielectric fluid as a primary insulation in a transformer impacts its design, reliability, and performance. Insulating and thermal characteristics of dielectric fluid mostly comprehend power equipment such as transformers, circuit breakers, and their functional limits and its optimal sizes. Most of the conventional insulating fluids suffer from lower cooling performance and limited dielectric capability [[1], [2], [3]]. It also restricts the further enhancement of the transformer's performance, as higher temperatures lead to irreversible impairment in the transformer's insulation. Many vegetable fluids, as well as synthetic fluids, are introduced as insulating fluids to achieve better dielectric and thermal performance [4,5].

The insulating oil in power equipment such as the transformer is bi-functional; it acts as an insulation between the conducting elements and also works as coolant by eliminating the heat generated during the operation. Even weight, size, and current density of the windings of the transformer depending on the rate of heat transfer and quantified volume of the insulating oil [6]. Most of the insulating oil shows the reduction in breakdown voltage over time that is one of the major issues during the aging of insulating oil [7]. In addition to the insulating particles (paper and silica), semiconducting particles (carbon), and conducting particles in insulating is studied to analyze their impact over time [8].

Nano based dielectric fluids, or in short, “Nanodielectric Fluids” (NDF) are colloidal solutions prepared by the uniform and stable dispersion of nanoparticles (NPs) in insulating oil and act as dielectric fluid [9]. Initially, conducting NP was utilized to enhance the properties of conventional oils with appropriate dispersion. Many conducting particles including Fe_3_O_4_, CuO, ZnO, Fe_3_P are utilized to prepare nano-oil and investigated thermal, dielectric, magnetic and chemical properties of the nano-oil [[10], [11], [12]].

The stability of NP is crucial in nano-based oil, which is attained by adding surfactants depending on colloidal components, NP, and insulating oil [4]. The magnetic nature of conducting NPs suffers from quick aging and agglomerates easily under an electric field [13].

**Fig. 1:**
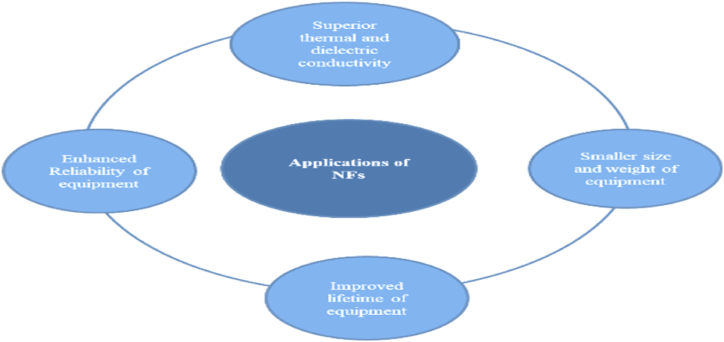
Nanofluids Benefits that enhance its applications.

Semiconductor NPs have similar behavior close to metals and non-metals. These NPs have wide band gaps, resulting in distinct properties when tuned. Some examples are Silicon, Germanium, GaN, CdS, GaP, and CdTe [14]. Nano-TiO_2_, a nano-semiconducting material used in mineral oil, results in enhancement of breakdown voltage [15]. With a 1% increment in concentration of TiO_2_ NP, it is noted to have an increment in breakdown voltage by 1.15–1.43 times as compared to the pure base oil. The surface modification of nano-TiO_2_ using octadecanoic acid improves the dielectric properties and breakdown voltage [16]. TiO_2_ NPs also show improvement in AC, DC and lightning impulse breakdown voltage with mineral oil and ester oil [17,18]. SiO_2_ NP based insulating oil is investigated and shows an increment in dielectric characteristics along with a comparison with magnetic NP based oil [19]. Al_2_O_3_NP shows 35% increment in AC breakdown voltage (AC BDV) of synthetic ester oil and enhancement in impulse breakdown strength [20,21]. Muhammad et al. [22] analyzed and compared the modified and unmodified oils using SiO_2_nanoparticles, which gave a higher AC breakdown voltage in the presence of moisture content for the modified oil. Prasath et al. [23] analyzed the modified MO (Mineral oil) with CaCu_3_Ti_4_O_12_ (CCTO) nanoparticles, which results in a higher dielectric constant and increment in breakdown strength. Víctor et al. [24,25] examines how different nanodielectric fluids made by dispersing different quantities of Fe_3_O_4_ nanoparticles in a mineral oil improved the AC breakdown voltage. The effect of semi-conductive SiC(Silicon Carbide) nanoparticles on the breakdown strength and partial discharge generation in natural ester oil FR3 has been examined [26]. Recently, Muzafar et al. [27] and Mehmet et al. [28] covered a number of research articles that thoroughly examined the performance of NDF, including electrical breakdown voltage, impulse test, dielectric, and thermal behaviour. These studies encompass the creation of various types of nanomaterials and their synthesis. Hence, NDFs conveniently meet all the conditions for being a dielectric fluid, as shown in Fig. 1. Although many NDF developed with their advantages and disadvantages, still nano infusion in dielectric fluids is in progress. It should have efficient electrical and thermal characteristics, reliable operation, and improved performance, which reduces equipment size and results in a longer equipment lifespan. This research work investigates the performance of a newly developed NDF.

In this paper, a novelCerium Oxide (CeO_2_) NP has been suspended into different types of insulating oils to study its effect on various dielectric properties enhancement. The characterization of CeO_2_ nanoparticles was studied using the FTIR (Fourier Transform Infrared) spectrum, the XRD (X-ray Diffraction) spectrum, the SEM (Scanning Electron Microscopy), and the TEM (Transmission Electron Microscopy). The nanofluid has been prepared using two step method. The various dielectric properties measured for pure oils and nanofluids are AC breakdown voltage and dielectric constant. For commercial mineral oil, real time aging has also been performed, and its change in dielectric properties has been measured over time. The important dielectric properties measured for aged mineral oil and its nanofluid are breakdown strength, dielectric constant, and dielectric constant. The statistical and predictive analysis has been performed on Statistics and Machine Learning Toolbox in MATLAB. The enhancement in breakdown voltages is found to be about 30% for mineral oil at 0.01 g/L nanoparticle concentration, 9% for synthetic ester oil at 0.03 g/L, 18% for rapeseed oil at 0.02 g/L, and 19% for soybean oil at 0.03 g/L nanoparticle concentration. Following the dispersion of CeO_2_ nanoparticles, the dielectric constant of all insulating oils has significantly improved.

## 2 Experimental details and procedure

The properties of different dielectric fluids in which NP is dispersed are discussed. The properties of the nanoparticle and surfactant used in this work are discussed in detail in the section below. The characterization of nanoparticles is done, and the preparation of NDF is explained in sections 2.2 and 2.3 respectively.

### 2.1 Materials used

Table 1 shows the basic properties of the insulating oils used in this paper. Cerium oxide nanoparticle used in this experiment was purchased from Platonic Nanotech Pvt. Ltd., India, for the investigation of its characteristics in insulating oil based on dielectric performance. The properties of nanoparticles and surfactants are listed in the Table 2 and Table 3, respectively.

**Table 1:** Properties of dielectric fluids.

Properties	Mineral Oil	Rapeseed Oil	Synthetic Ester Oil	Soybean Oil
Density (at 40 °C) (kg/dm^3^)	0.859	0.89	0.94	0.89
Kinematic viscosity (*at 40°C*) (mm^2^/s)	7.63	35	28	30
Flashpoint (°C)	145	315	288	315
Fire point (°C)	164	340	330	338
Pour point (°C)	−26.5	−31	−56	−18
Ph	7.08	20.2	20.9	22.0

**Table 2:** Properties of nanoparticles.

Properties	(CeO_2_)
Assay	99.90%
Particle size	20–30 nm
Surface area	40–50 m^2^/g
Melting point	2400 °C
Density	6.5 g/cm^3^

**Table 3:** Properties of surfactant.

Properties	Cetyl Trimethyl Ammonium Bromide (CTAB)
Description	Cationic
Form	Solid
Color	White
Melting point (°C)	248–251
Flash point (°C)	244
Flammability	Not flammable

### 2.2 Characterization of CeO_2_Nanoparticle

**Fig. 2:**
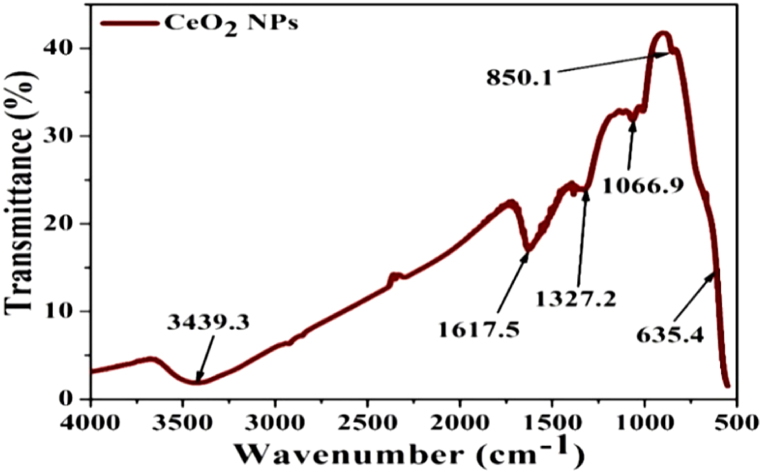
FTIR spectra of CeO_2_ NPs.

**Fig. 3:**
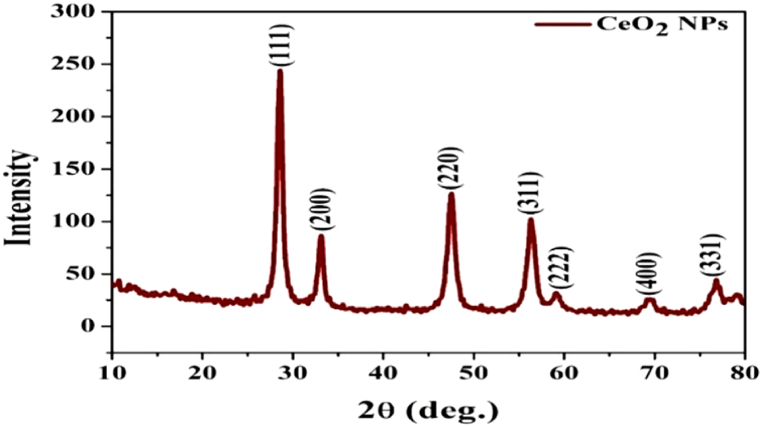
XRD spectrum of CeO_2_ NPs.

The FTIR (Fourier transform infrared) spectra of CeO_2_ NP is shown in Fig. 2. The spectra were recorded by the Perkin-Elmer-1725X instrument on Potassium Bromide (KBr) pellets between the wave number of 4000-400 cm^−1^. In the spectrum of CeO_2_ NPs, peaks were detected at 3439.3 cm^−1^ and 1617.5 cm^−1^ which may be because of the O–H stretching vibration of the water molecule. The peaks at 1327.2 cm^−1^ and 1066.9 cm^−1^ are attributed to the carbonate like species formation on the surface of CeO_2_ NPs. The peak at 850.1 cm^−1^ is owing to envelopment of the phonon band of CeO_2_ NPs. The broad peak at about 635.4 cm^−1^ might be due to the vibrational mode of O–Ce–O bonds [[29], [30], [31]]. The XRD (X-ray diffraction) spectrum of CeO_2_ NP is given in Fig. 3. The spectrum was obtained at room temperature by a Bruker D8 diffractometer with CuKα radiation at 1.540 Å in the range of 2θ = 20°–80° at 50 kV. The scan rate was kept at 0.02° s^−1^.

The XRD spectrum shows sharp and high-intensity peaks, revealing the highly crystalline nature of the CeO_2_ NPs. The characteristic peaks of CeO_2_ NPs are observed at 2θ = 28.62°, 33.14°, 47.58°, 56.24°, 58.96°, 69.60°, and 76.92°, which may be indexedrespectively as (111), (200), (220), (311), (222), (400) and (331) crystalline planes of CeO_2_ NPs [[29], [30], [31], [32]].

**Fig. 4:**
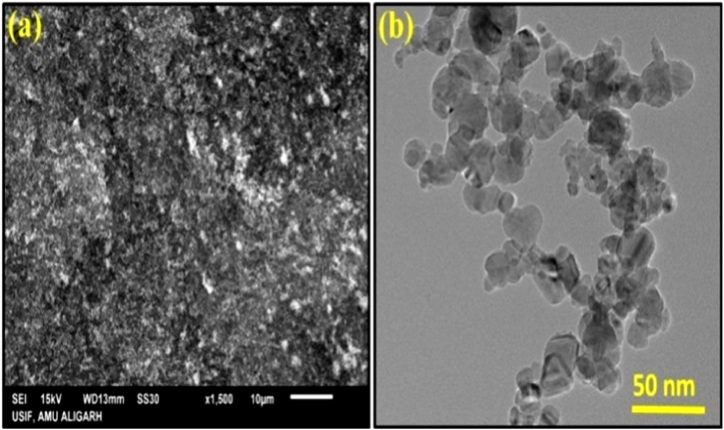
**(a)** SEM and **(b)** TEM image of CeO_2_ NPs.

The morphology, as well as structure of CeO_2_ NPs, are studied by SEM (Scanning Electron Microscopy) and TEM (Transmission Electron Microscopy) techniques. The SEM and TEM images of CeO_2_ NPs are shown in Fig. 4.

The SEM image was obtained through the gold coating on the surface of CeO_2_ NPs by JEOL, JSM 6510-LV (Japan) scanning electron microscope instrument. The TEM image was recorded by JEM 2100, JOEL (Japan) transmittance electron microscope instrument. The SEM image of CeO_2_ NPs (Fig. 4(a)) shows that the surface of CeO_2_ NPs consists of agglomerated nanoparticles forming a rough surface. The TEM image (Fig. 4(b)) provides clear evidence of the semi-spherical shape and size of CeO_2_ NPs. A large number of semi-spherical nanoparticles are seen in the TEM image, which are connected to each other.

### 2.3 Preparation of nano based insulating oil

**Fig. 5:**
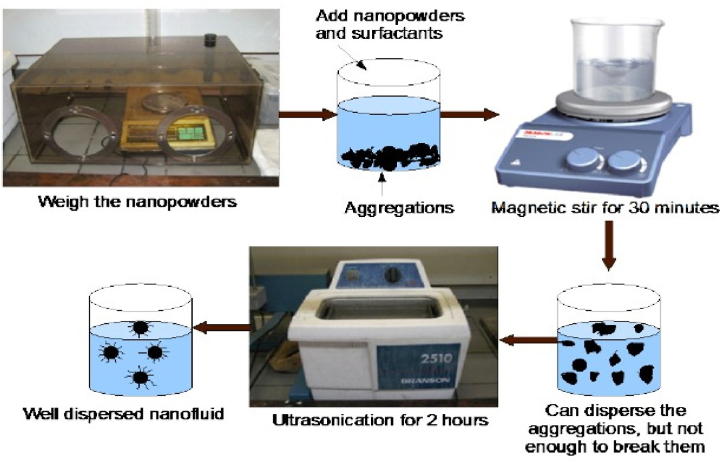
Two step method for preparation of NDF.

The nanoparticles are non-soluble compounds that form a stable colloid known as nano dielectric fluid (NDF) when dispersed in the insulating oil. Normally, two different methods are used for the preparation of NDF; One-step method and Two-step method. The one-step method includes synchronous synthesis and suspension of NP into insulating oil, i.e., the removal of water, depoting, and shipment of nanoparticles are not to be noticed, resulting in decreasing of agglomeration and improvement in stability of NDF [14]. Two step method consists of two steps. The former step is the preparation of nanofiller like nanotubes, nanoparticles as powder, and the latter step is the dispersion of NP into insulating oil using a magnetic stirrer and an ultrasonicator, as shown in Fig. (5).

The Two-step method is more preferred over the One-step method because of its lower cost, less wastage, and no additional or specialized equipment implementation to produce in bulk. Mostly, a surfactant is added to insulating oil that acts as a stabilizing reagent. Its objective is to minimize the repulsive forces between the NPs and provide stable dispersion of NP in insulating oil for a longer lifespan. It has likewise been accounted for that the expansion of a lot of surfactants can diminish the dispersal time and corrupt the dielectric properties of NDF [14,28].

In this paper, CeO_2_-nanoparticles-based nanofluids are prepared using the two-step method. Initially, the surfactant Cetyl Trimethyl Ammonium Bromide (CTAB) in an amount equivalent to 50% of the volume concentration of nanoparticles is added to insulating oil to ensure stable dispersion of NP. The concentration of CTAB under study is 50% of the volume concentration of nanoparticles. It is based on the similar study in Ref. [38] where the optimum concentration of surfactants is found at that concentration level for different type of insulating oils.

The accurate mixing of surfactant in insulating oil is attained by using a magnetic stirrer for 30 min. Then CeO_2_ is being added to the prepared solution. To ensure uniform mixing, ultrasonication is done on the solution for 2hrs. After ultrasonic mixing, the solution was kept at room temperature for 24 h to permit the arrival of the air pockets produced by the ultrasonication, and lastly, the solution was put in a heating oven where they were dried and degasified at 60 °C and 0.1 atm for 24hrs. The prepared solution was kept in sealed vials at ambient temperature to test long-term stabilization. This work includes various concentrations of CeO_2_ NP, i.e., 0.005, 0.01,0.02, and 0.03 g/L, that are added to form nano-oil sample.

## 3 Measurement

The introduction of nanotechnology provides advancement to attain better insulating oil with improved critical parameters that make it possible to operate for longer periods, with less cost and maintenance. Aging is also a crucial factor required to analyze any insulating oil. In this paper, dielectric strength, and dielectric properties have been measured and discussed further in the section below:

### 3.1 Dielectric strength

To implement the oil insulation in the transformer, the AC BDV is one of the important preconditioning parameters. It is affected by contaminants like tiny particulates, moisture, and air or gaseous bubbles. The obtained AC BDV indicates the quality of the oil. Lightning impulse breakdown voltage (LI BDV) is generally examined by IEC 60897 standards. Various factors of NPs, like their type, concentration, and shape, affect the LI BDV.

In this work, measurement and experimentation was conducted on pure insulating oils and its nanofluid to analyze breakdown voltage with different concentration of nanoparticles. The breakdown is explained using three models, as follows: (i) Bubble formation at the surface of the electrode, where bubbles are generated due to local heating and field-emitted electrons injected into the gap among the electrodes, (ii) Trapping and de-trapping of electrons, which includes electron-hopping transport in the traps and transport in a delocalized state: and (iii) Streamer propagation, Ionization of molecules due to a dependent electric field is a direct ionization mechanism, where a huge electric field results in the extraction of an electron from a neutral molecule, thus generating, a free positive ion and a free electron. In this experiment, CeO_2_ semi-conductive nanoparticles with different concentrations are suspended in different insulating oils, and their corresponding breakdown voltages are analyzed. It was noted that breakdown voltage increased with incorporation of NPs. The explanation of steps for increased breakdown voltages is as follows:

(1) NPs are polarizable. The formation of bubbles is affected due to semi-conductive nanoparticles, which cause an increment in breakdown voltage.
(2) The fast electrons are slowed down by electron trapping and de-trapping in shallow traps of nanofluids (NFs), resulting in improved breakdown performance as compared to that of pure oil.
(3) Reduction in molecular ionization in transformer oil because of conductive semi-conductive nanoparticles. It slows down the electron and hence hinders the streamer propagation. It also considers electron scavengers.

**Fig. 6:**
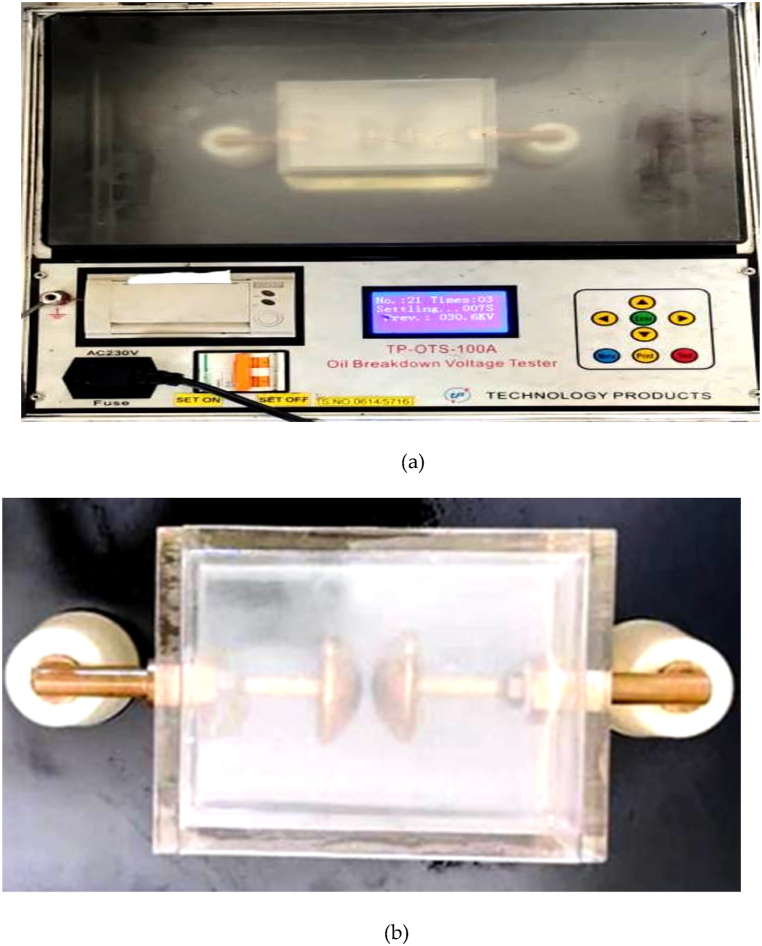
(a) Breakdown voltage tester (b) Oil cell with the gold-plated mushroom electrodes.

Oil breakdown voltage tester model TP-OTS-100A (Technology Products, New Delhi), as shown in Fig. 6(a) used to study the breakdown voltage (BDV) of oil samples [34]. It applies a 0–230 kV AC voltage with a 2 kV/s ramp rate. The mushroom electrodes are used to measure breakdown voltage for all the reported samples as shown in Fig. 6(b). The electrodes are gold plated of shape mushroom and the gap between them is fixed at 2 mm as per IS 6792, IEC 60156 [35,36]. After positioning the sample and distance setting, a 10-min wait was given before running the test. Two minutes between each measure and the next are respected for all samples; each sample consists of twelve measurements. The obtained AC-BDV data were recorded and then analyzed using Weibull distribution and plotted in form of box and whisker plots. All NF samples are prepared and tested for each concentration.

### 3.2 Dielectric constant

An insulating oil acts as a medium that separates electrodes or ground from each other in an electrical network. It can act alone as a medium or maybe combined with other insulating materials. It functions as a dielectric for the capacitor. The small value of the dielectric constant of the oil indicates small capacitance. This small value remains stable overheat transfer and other chemical properties. The intermediate value of permittivity may be an advantage for achieving better voltage distribution with oil in series. Testing may be conducted as per various standards like IEC 60247, ASTM D924, and IS 6262/6103 [37].

### 3.3 Aged condition

Aging is a crucial parameter to indicate deterioration in the oil. The parameter shows electrical properties. It also affects the normal functioning and life of high voltage devices like transformers. Sometimes it may fail. In the process of aging of oil, by-products are released like acids, and hence the neutralization value increases [33].

## 4 Results & discussion

The development of a stable and reliable dielectric fluid is crucial for transformer performance and operation. In this work, we developed different nano oil using different base oils with varying nanoparticle concentrations. A comparative study is necessary and is done by analyzing the dielectric properties of the prepared nanofluids. This section summarizes the alteration in properties of insulating oil in presence of a different concentration of CeO_2_ nanoparticle. As per the standard procedure [34] utilized for insulating oils, an iterative process of breakdown measurement is performed on each sample of dielectric oil to ensure the obtained breakdown value has a specific range along with the average value.

### 4.1 Breakdown strength

**Fig. 7:**
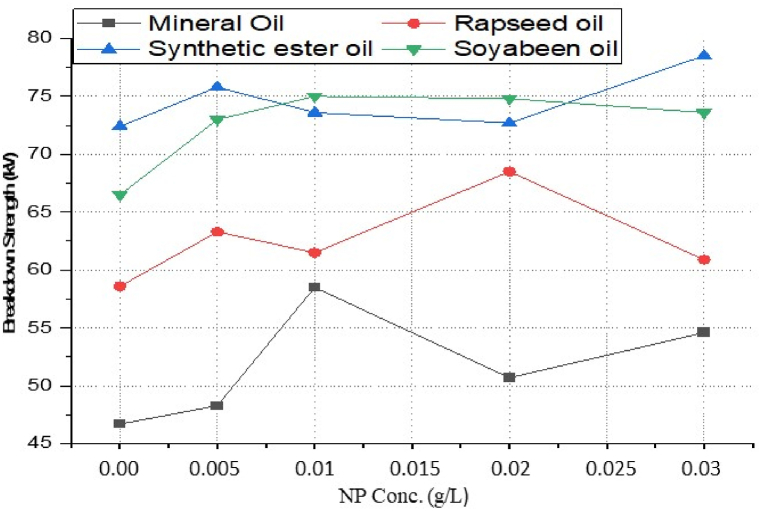
Average breakdown strength of different insulating oil-based nano-oil at different nanoparticle concentration.

The AC breakdown voltage is a key indicator of the electrical strength of the insulating oil; it indicates the high-voltage resistance in electrical equipment. Fig. 7 shows the average AC breakdown voltage of four different oils with different volume fractions of CeO_2_ nanoparticles. The nano-oil shows the change in the dielectric strength of base oil with the amount of added nanoparticles. Four different types of oil consisting Mineral oil, Rapeseed oil, Synthetic ester oil and Soybean oil. All used oils are pure and fresh to ensure the change in characteristics only depend on dispersed nanoparticles. The mean breakdown voltage of nano oil with single base oil shows the increment with an increase in nanoparticle concentration. The trend is almost similar for a small additive amount, which later on decreases due to a dispersion limit and possible agglomeration beyond the saturation point. Mean breakdown voltage shows the change in nano-oil all together but sometimes does not exactly comprehend the scenario.

**Fig. 8:**
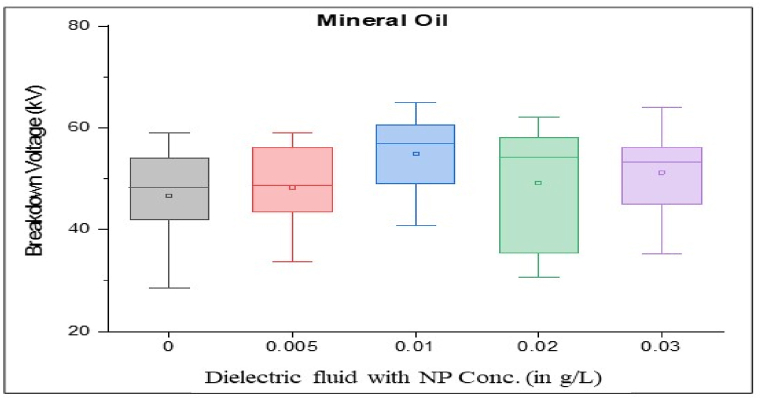
Box and whisker plot representation of breakdown strength of Mineral Oil based nanofluids.

**Fig. 9:**
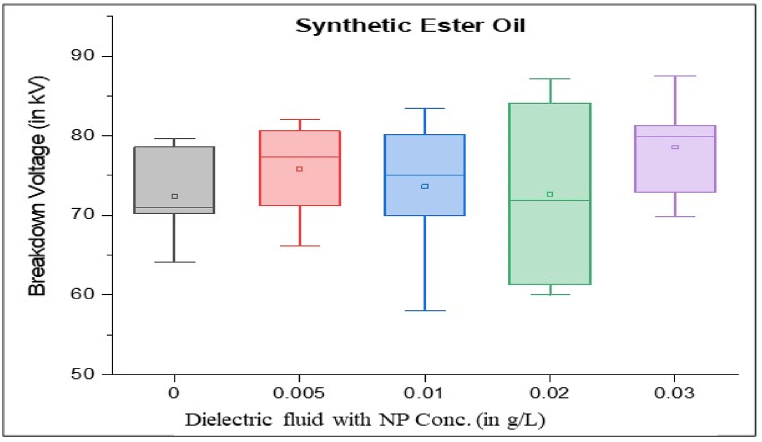
Box and whisker plot representation of breakdown strength of Synthetic ester Oil based nanofluids.

**Fig. 10:**
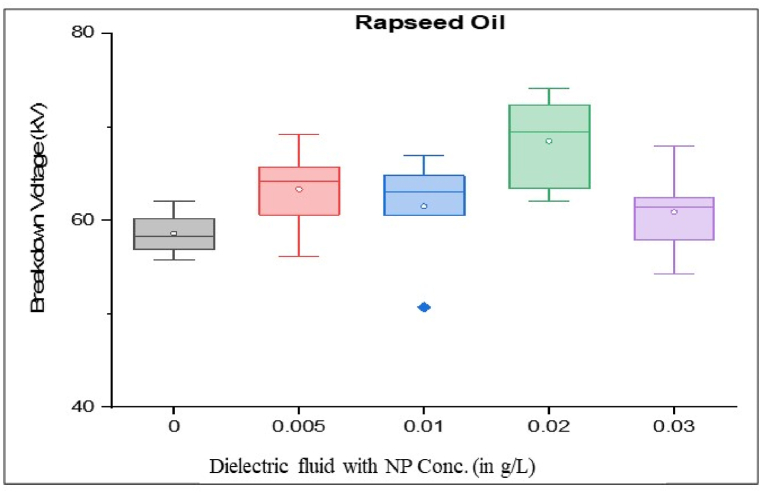
Box and whisker plot representation of breakdown strength of Rapeseed Oil based nanofluids.

**Fig. 11:**
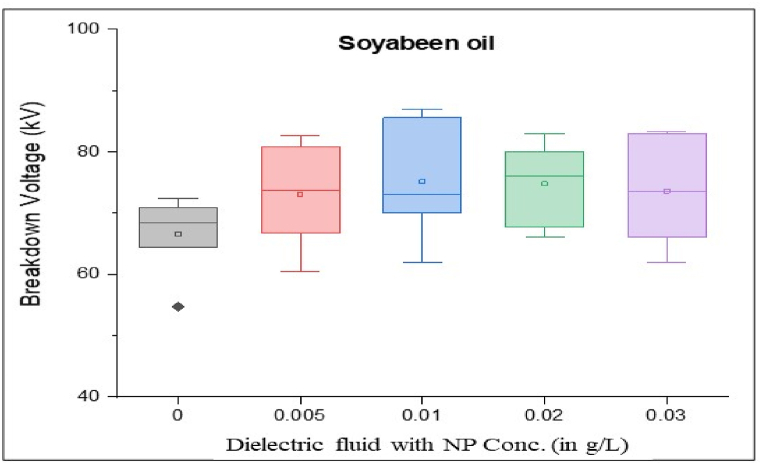
Box and whisker plot representation of breakdown strength of Soybean Oil based nanofluids.

As per standard guidance [34], insulating oil goes under multiple breakdown tests to minimize error in measured breakdown voltage. Failure in the insulating oil cannot rely on mean breakdown voltage. It should be done by considering all aspects, such as producing a box and whisker plot for each sample. Iterative breakdown voltage results can be plotted, consisting of mean, median, range, 5th and 99th percentiles represent collectively in the form of a box and whisker plot as shown in Fig. 8, Fig. 9, Fig. 10, Fig. 11.

Box and whisker plots for different nanoparticle concentrations are formed explicitly for each base oil. Mineral oil is the most commercially used dielectric fluid for transformers and other oil-filled insulation systems. Fig. 8 represents box and whisker plots of breakdown voltage for different NP concentrations dispersed in mineral oil. Boxes represent the AC breakdown voltage of pure oil and its nanofluid with different concentrations of nanoparticles, i.e., 0.005, 0.01, 0.02, and 0.03 g/L dispersed in mineral oil. The maximum breakdown voltage is achieved with 0.01 g/L nanoparticle concentration. The Box and whisker plot for 0.02 g/L shows the maximum variation in breakdown voltage, which increases the failure possibility.

Fig. 9 shows the box and whisker plot representation for the breakdown voltage of the nano oil sample taking synthetic ester oil as the base oil. Although the highest breakdown voltage was achieved with 0.03 g/L NP concentration but the average value is lower than the maximum voltage. The most variation in breakdown voltage occurs at 0.02 g/L NP. Fig.10shows the box and whisker plot representation of nano-oil with rapeseed oil as the base. Most breakdowns show minimum variation except for 0.01 g/L NP and maximum breakdown voltage achieved at 0.02 g/L concentration. Breakdown voltage as a box and whisker plot for soybean based nano oils is shown in Fig. 11. The highest breakdown voltage is achieved at 0.01 g/L concentration.

### 4.2 Investigation of breakdown voltage without surfactant

The addition of surfactants brings several advantages to nanofluids, making them more suitable for enhancement of dielectric properties. Here are some key advantages of using surfactants in nanofluids: (a) Improved stability: Nanoparticles tend to agglomerate and settle down in the base fluid, leading to poor stability. Surfactants act as stabilizing agents by adsorbing onto the nanoparticle surface, preventing agglomeration and maintaining uniform dispersion. This improves the long-term stability of nanofluids. (b) Controlled particle size distribution: Surfactants can influence the aggregation and dispersion behavior of nanoparticles, allowing for the control of particle size distribution in nanofluids. This control is crucial for achieving desired properties and optimizing the performance of nanofluids in specific applications. (c) Compatibility with base fluids: Surfactants can be selected and tailored to be compatible with different types of base fluids, enabling the formulation of nanofluids with a wide range of properties. This versatility makes nanofluids more adaptable to various industrial processes and heat transfer systems.

**Fig. 12:**
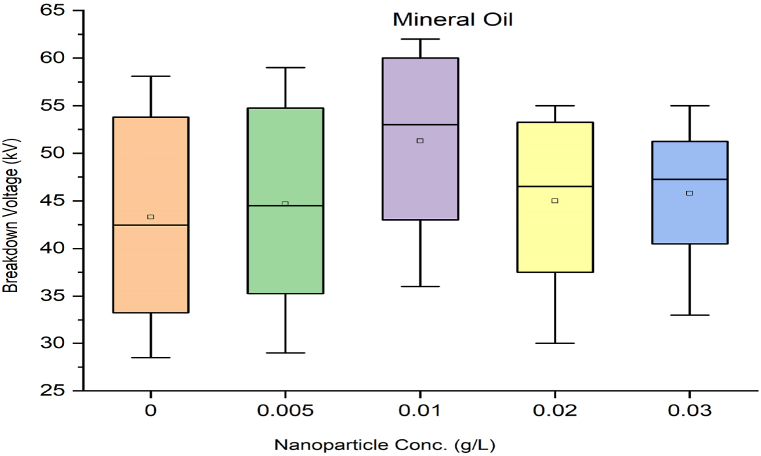
Box and whisker plot representation of breakdown strength of Mineral Oil based nanofluids without surfactant.

**Fig. 13:**
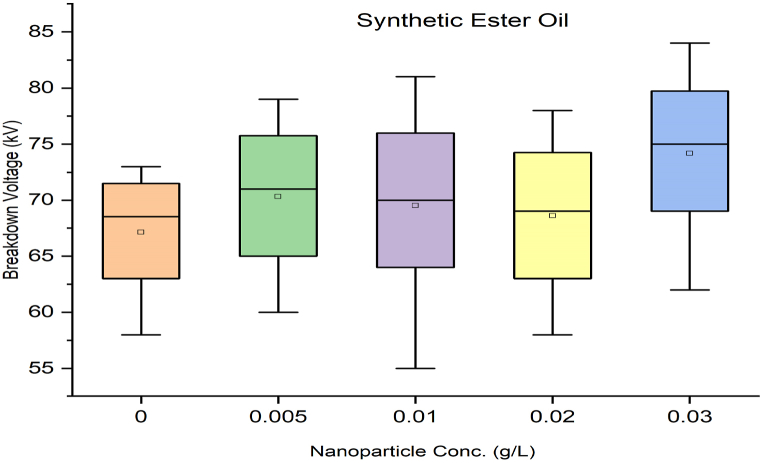
Box and whisker plot representation of breakdown strength of Synthetic Ester Oil based nanofluids without surfactant.

**Fig. 14:**
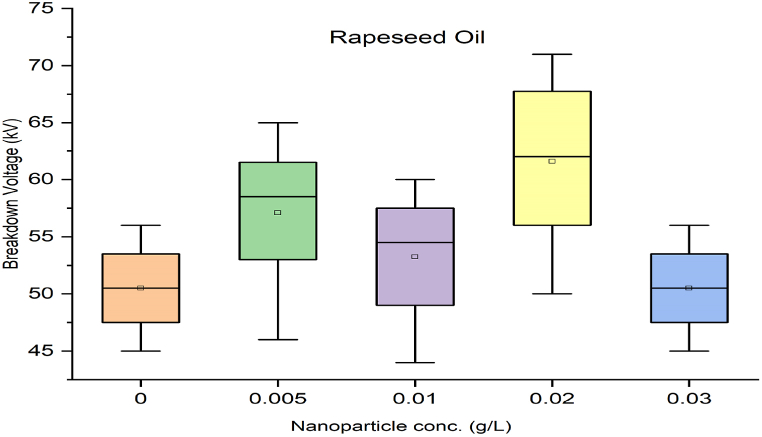
Box and whisker plot representation of breakdown strength of Rapeseed Oil based nanofluids without surfactant.

**Fig. 15:**
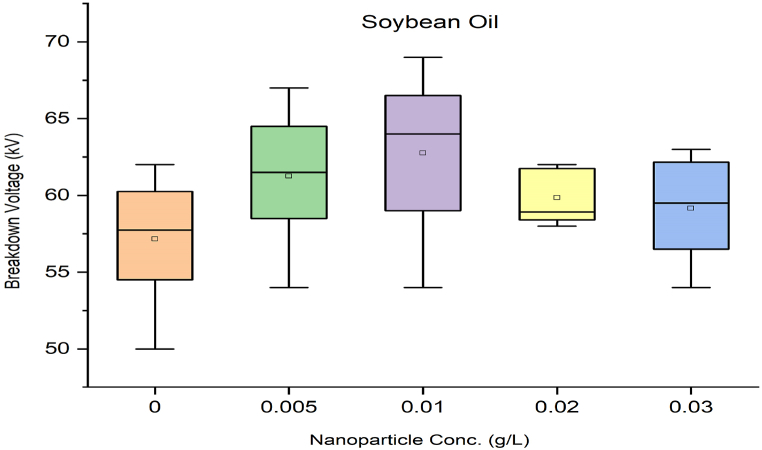
Box and whisker plot representation of breakdown strength of Soybean Oil based nanofluids without surfactant.

Fig. 12, Fig. 13, Fig. 14, Fig. 15 show the box and whisker plot representations for the breakdown voltage of the different nano samples in mineral oil, synthetic ester oil, rapeseed oil, and soybean oil as the base oil without surfactant, respectively. It is observed that the overall mean breakdown voltage is slightly reduced as compared to when surfactant CTAB was used. The possible reason for this is that the nanoparticles agglomerate, leading to non-uniform dispersion in the above insulating oils. This results in areas of high electric field concentration, which decreases the breakdown strength of the oils.

### 4.3 Statistical analysis of breakdown strength using Statistics and Machine Learning Toolbox

**Fig. 16:**
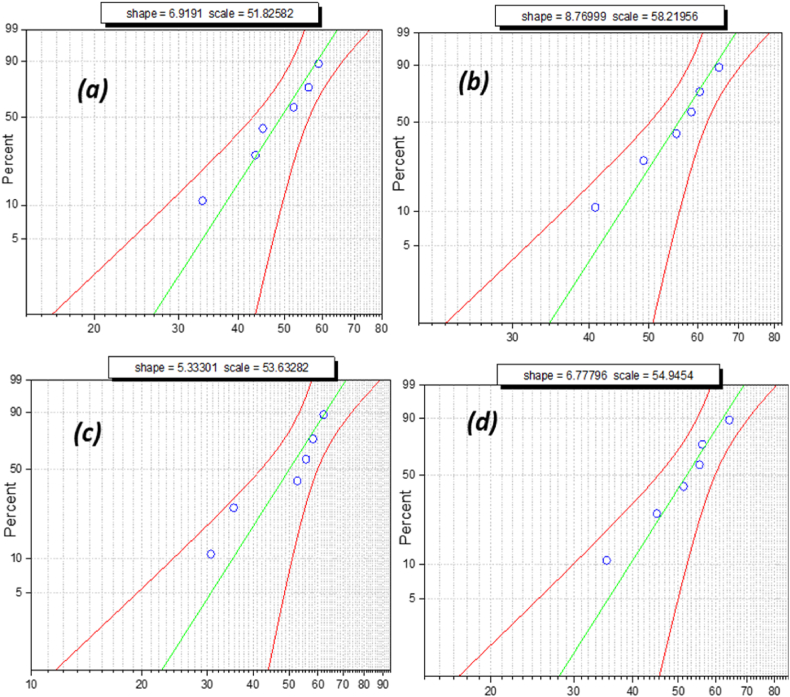
Weibull distribution plots for nano-oil with mineral oil as base fluid with different nanoparticle concentration. (a)0.005 g/L (b) 0.01 g/L (c) 0.02 g/L (d) 0.03 g/L.

**Fig. 17:**
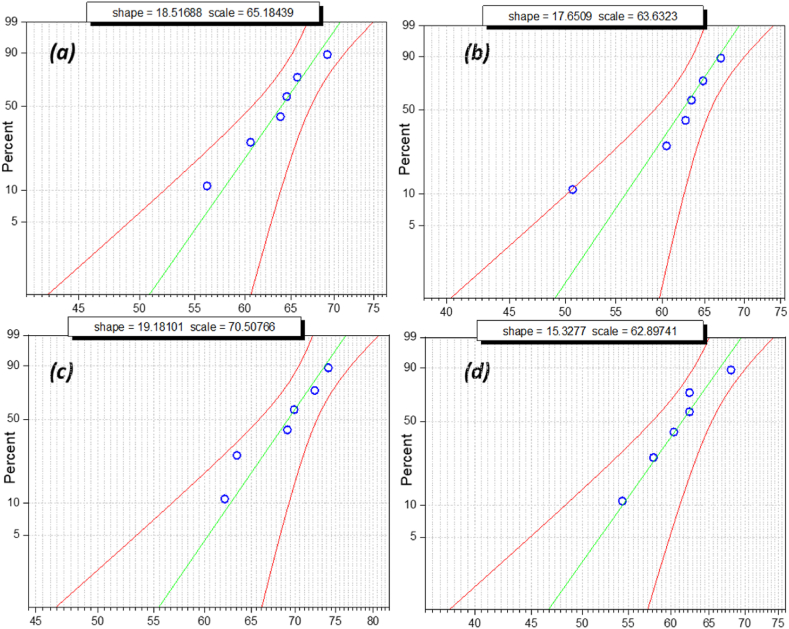
Weibull distribution plots for nano-oil with Rapeseed oil as base fluid with different nanoparticle concentration. (a)0.005 g/L (b) 0.01 g/L(c) 0.02 g/L (d) 0.03 g/L.

**Fig. 18:**
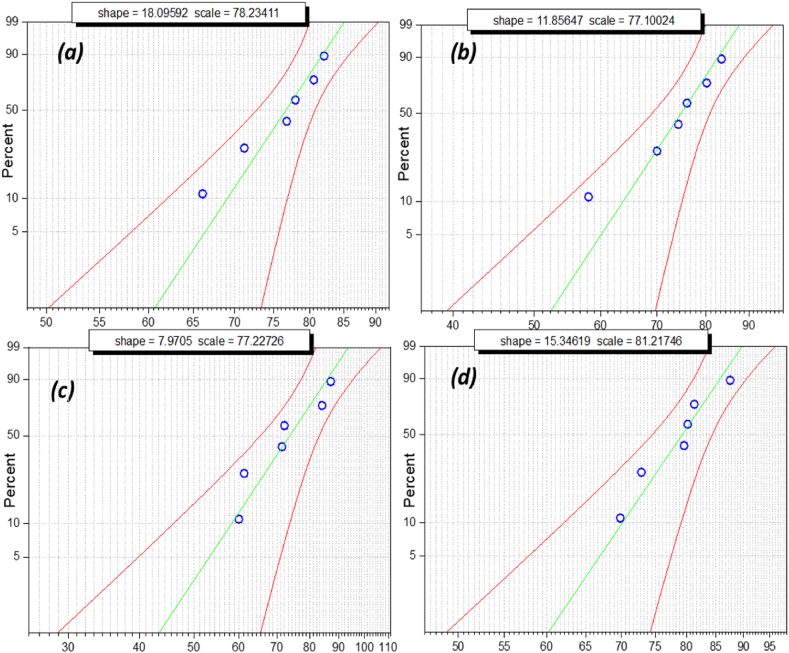
Weibull distribution plots for nano-oil with synthetic ester oil as base fluid with different nanoparticle concentration. (a)0.005 g/L (b) 0.01 g/L (c) 0.02 g/L (d) 0.03 g/L.

**Fig. 19:**
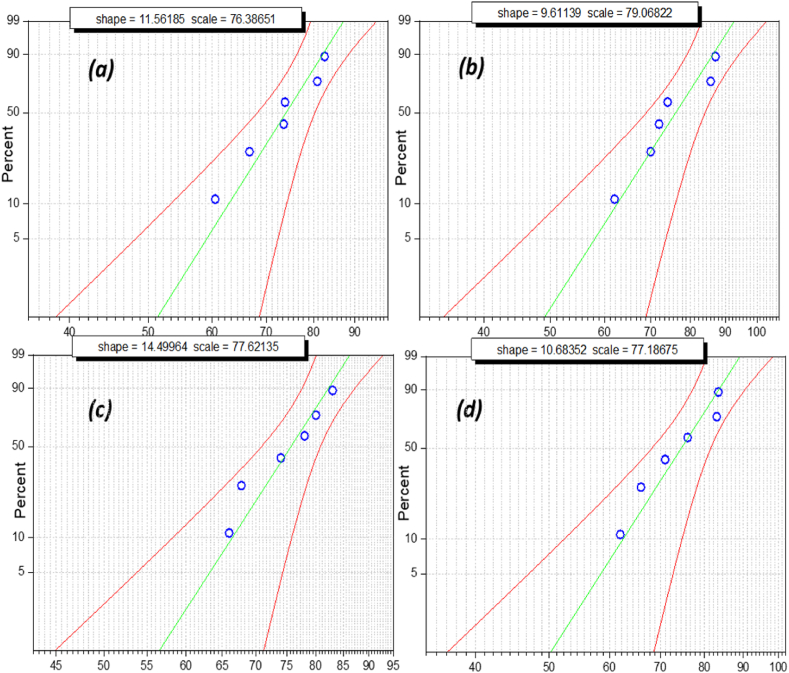
Weibull distribution plots for nano-oil with Soybean oil as base fluid with different nanoparticle concentration. (a)0.005 g/L (b) 0.01 g/L (c) 0.02 g/L (d) 0.03 g/L.

The above box and whisker plots show the normal distribution of breakdown voltage, specifically when nano-oil reaches its maximum strength. Failure possibility cannot be determined by the mean or average value of breakdown voltage. Failure possibility is an estimate in terms of probability distribution to ensure dielectric fluid workability. Weibull distribution is a significant tool for measuring its reliability as an insulating liquid in electric assets such as transformers and circuit breakers. Weibull distribution analysis is utilized to estimate the failure possibility of oil samples using iterative breakdown voltage. The general expression of the 2-parameter Weibull distribution is as follows:

(1) $$P\left(a\right)=1-E^{\left\{-{\left(BDV/\eta \right)}^{\alpha }\right\}}$$

where, *P(a)* is the cumulative probability density of breakdown; BDV is the breakdown voltage; α is the shape parameter, and***η*** is the scale parameter. The obtained experimental data sets were analyzed using the Statistics and Machine Learning Toolbox in MATLAB. The Regression and Classification algorithm in this toolbox was used to predict the probability of failure, as shown in the Weibull probability distribution plot below Fig. 16, Fig. 17, Fig. 18, Fig. 19. The Weibull probability distributions plot shows the probability of breakdown (%)with respect to the AC breakdown voltage (kV) of nano-oil based on mineral oil, rapeseed oil, synthetic ester oil, and soybean oil, respectively, with different nanoparticle concentrations. The green line in Fig. 16, Fig. 17, Fig. 18, Fig. 19 represents the regression line or prediction line, and redlines represent the 95% confidence interval.

Shape and scale factor is provided in each distribution plot for every nano-oil with different concentration of CeO_2_ NPs. The Weibull distribution plot for mineral oil shown in Fig. 16(a–d) for different concentration of nanoparticles, it can be observed that for increase in breakdown voltage the failure probability also increases and there is a small deviation from prediction line. For Rapeseed oil in Weibull distribution plot, Fig. 17(a–d) there has been an increase in both shape and scale factor. Also, the 95% confidence interval has a wide range and the maximum deviation can be observed for 0.01 g/L CeO_2_ nanoparticle concentration. The Weibull distribution plot for synthetic ester oil has been shown in Fig. 18(a–d) for all used CeO_2_ nanoparticle concentration. It shows minimum deviation for 0.01 g/L concentration. The Weibull distribution plot for soyabean oil, Fig. 19(a–d) shows that lower breakdown voltage has lower 50% failure probability and maximum deviation from prediction line is for 0.005 g/L CeO_2_ nanoparticle concentration. From Fig. 16, Fig. 17, Fig. 18, Fig. 19, we can observe that as nanoparticle concentration is increased, the 50% failure probability also increases for higher breakdown voltages. The CeO_2_ nanoparticles exhibited high insulation characteristics that lead to the improved breakdown voltage of the nano oil (NO). Charge accumulation at the surface of nanoparticles, due to polarization under electric field, delays the streamer's formation. Hence, the threshold electric field required for the breakdown is higher than the original electric field for base oil. As this charge trapping by CeO_2_ nanoparticles is better with NP concentration, thereby enhancing the overall breakdown condition for nano-oil.

### 4.4 Dielectric constant measurement

**Fig. 20:**
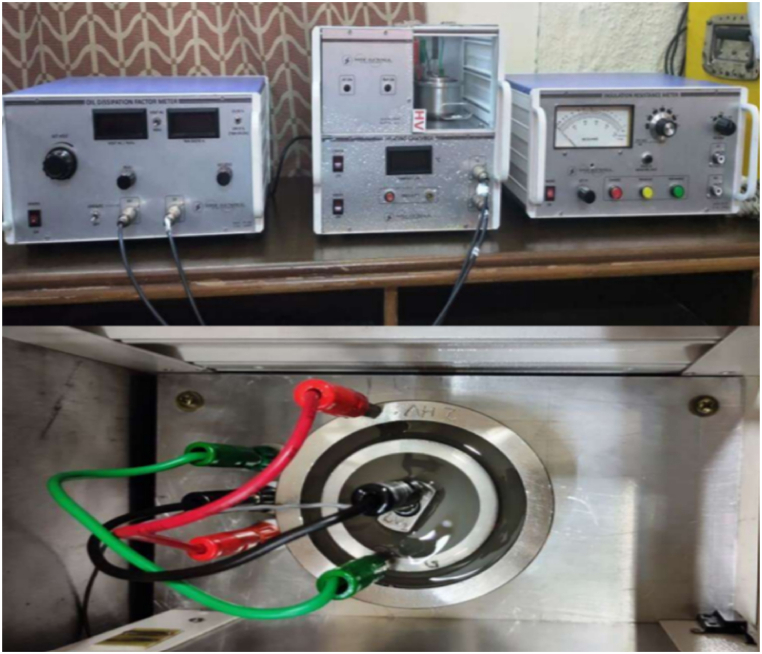
I. R Tester, Oil dissipation factor meter, heating chamber and test cell for measurement of dielectric constant.

The dielectric constant corresponds to the inability of dielectric molecules to reorient themselves with the alternating gradient of the electric field. Oil dissipation factor meter (model PE-ODF) which is employed to measure dielectric constant with AC voltage variation of 0–500 V output as shown in Fig. 20. Testing was conducted as per the following standards: IEC 60247, IS 6262/6103, and ASTM D924 [37]. After switching on the instrument, wait for 5 min for the stabilization, keep AC voltage at zero with the help of a set volt knob and gradually raise the voltage (up to 300 V) as desired to operate with the minimum stress. The dielectric constant measured ten times for each sample and the average reading was recorded.

**Fig. 21:**
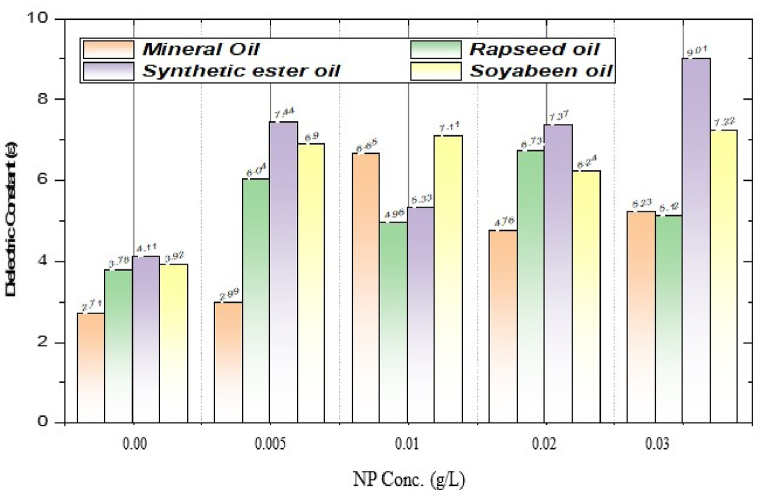
Dielectric constant of CeO_2_ based nanofluids at different concentrations of nanoparticle for different insulating oils.

Fig. 21 shows the plot of dielectric constant vs. nanoparticle concentration for different insulating oils used in the experiment. It can be observed that the dielectric constant enhanced significantly with the addition of CeO_2_ nanoparticles as compared to pure oil for all insulating oils used in this experiment. Nanoparticles absorb the electrons and decrease the oil molecules’ tendencies to align in direction of the electric field. Hence, the nano-oil is more polarized, and the dielectric constant increases. The increase in the dielectric constant is maximum at 0.01 g/L for mineral and soybean oil. Synthetic ester oil shows the maximum dielectric constant on adding nanofiller. Rapeseed oil has a maximum dielectric constant at 0.02 g/L of CeO_2_ nanoparticles.

#### 4.5 Aging measurement

Aging is an important parameter to comprehend the working condition of dielectric fluids under electrical, thermal, and chemical stresses with time. These unwanted stresses result in the degradation of dielectric fluid components by producing by-products that are unwanted contaminants and deteriorate the insulation characteristics. These contaminants reduce the electric field strength, shorten the life expectancy of dielectric fluid, and increase the chance of failure. Mineral oil is the most commonly used commercial insulating liquid, so in this experiment, the measurement of electrical properties with aging was done only on mineral oil and its nanofluid because of the unavailability of other base fluids in India, like synthetic ester oil, rapeseed oil, and soybean oil. In India, the electrical industry primarily follows standards and regulations that are based on the use of mineral oil as the preferred insulating oil. Consequently, it is not possible to obtain these real-time aged oils as they are not adopted or mandated by existing standards. Secondly, the cost of these oils is very high as compared to mineral oil; therefore, in our country, mineral oil is preferred in all types of transformers.

**Fig. 22:**
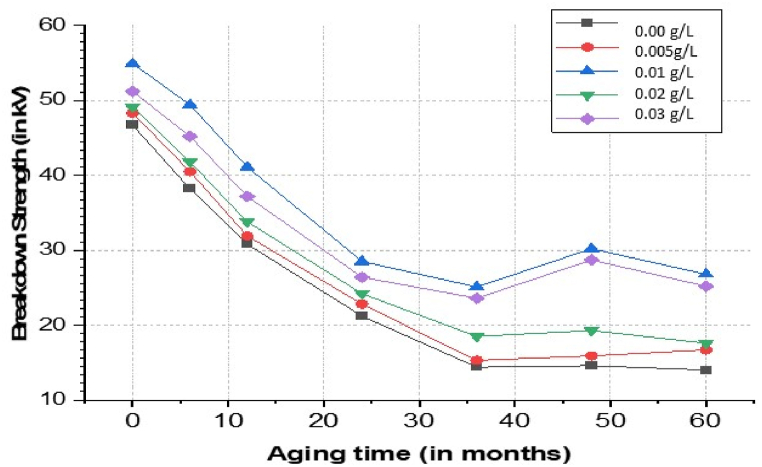
Breakdown voltage variation for mineral oil and its nano-based oil with respect to aging time.

Various properties such as the AC breakdown voltage, the dissipation factor, and the dielectric constant of oils were measured periodically. All these real time aged oils were obtained from 500 MVA oil immersed power transformer in successive durations. The range of time considered for measurement is 60 months. Fig. 22 shows the variation in breakdown voltage of pure mineral oil and its nanofluid using CeO_2_ nanoparticles in different concentrations over time. It can be observed that there is a significant reduction in breakdown strength of mineral oil and its nanofluid with aging. However, for nanoparticle concentrations of 0.01 g/L and 0.03 g/L the breakdown strength after 2 years is much better and more permissible as compared to pure mineral oil. Therefore, it can be concluded that the usage of CeO_2_ nanoparticles slows down the deterioration of mineral oil's breakdown strength with aging.

**Fig. 23:**
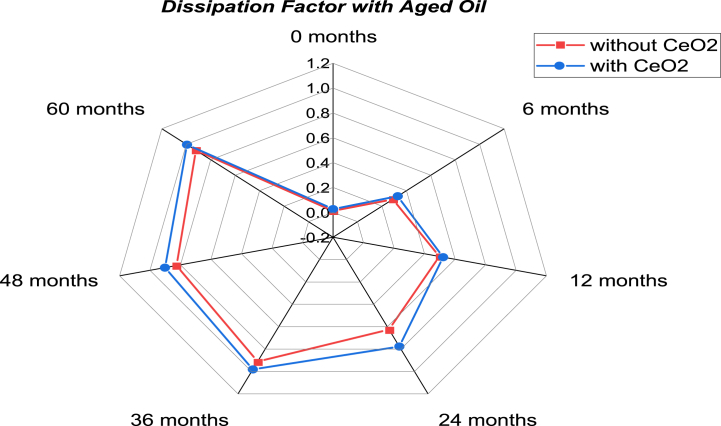
Dissipation factor for mineral oil and its nano-based oil with respect to aging time in months.

**Fig. 24:**
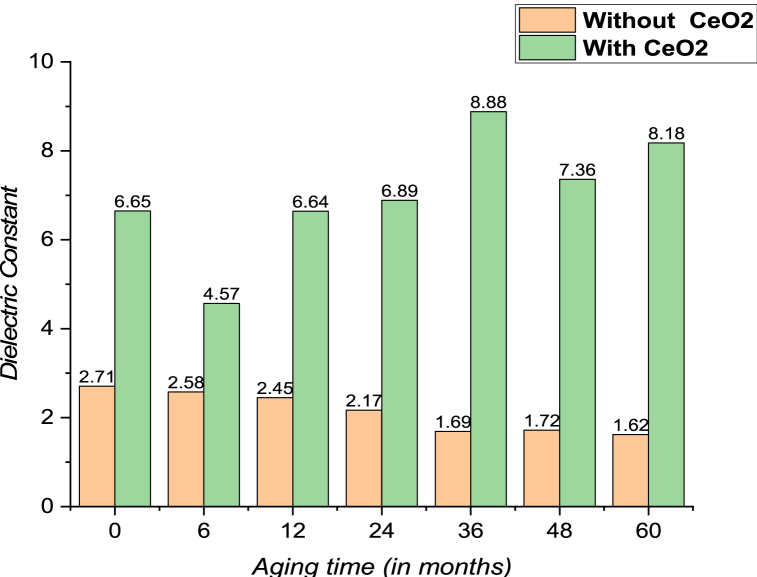
Dielectric constant for pure mineral oil and nano-based mineral oil with respect to aging time in months.

The dissipation factor and dielectric constant experimental data sets with aging were analyzed using predictive model of Statistics and Machine Learning Toolbox in MATLAB. The predictive model uses accelerated electrical and thermal stress aging and is found to have similar properties as those obtained in the experimental results. When an electrical insulating liquid is employed in an alternating electric field, the dissipation factor measures the dielectric losses (dissipated as heat). The dissipation factor grows with aging for both pure mineral oil and its nano-based oil, as shown in Fig. 23. Initially, the dissipation factor of pure mineral oil and its nano-based oil is nearly identical. The difference between the dissipation factor of nano oil and mineral becomes broader with aging time. The overall change in dissipation factor with aging is small for mineral oil and it's nano-based oil and does not impact the dielectric properties of aged mineral oil considerably. Fig. 24 shows the variation in dielectric constant in the presence of CeO_2_ NP dispersed in mineral oil over time. As shown in the figure, the dielectric constant of mineral oil decreases with aging, while nano-oil sustains the aging stresses efficiently. There is also an increase in dielectric constant with aging time due to the expansion of hydrocarbon compounds of mineral oil over aging, which increases the CeO_2_ absorption capability and improves the dielectric constant.

## 5 Conclusion

Implementation of nanotechnology in the area of dielectrics results in many research investigations. Nano based dielectric fluids provide many opportunities and increase their application. To draw a conclusion, an experimental investigation on the effect of CeO_2_ nanoparticles in different concentrations on the dielectric properties of various insulating oils such as mineral oil, rapeseed oil, synthetic ester oil, and soybean oil has been presented. The dielectric properties investigated are AC breakdown voltage, dielectric constant, and aging measurements. It has been observed that the breakdown voltage is enhanced by up to 30% for mineral oil at an optimal concentration of 0.01 g/L, 9% for synthetic ester oil at 0.03 g/L, 18% for rapeseed oil at 0.02 g/L, and 19% for soybean oil at 0.03 g/L nanoparticle concentration. Following the dispersion of CeO_2_ nanoparticles, the dielectric constant of all insulating oils has also significantly improved. The dielectric constant improved significantly as the concentration of NP dispersed in oil increased. Some results show a decrease with increasing NP concentration due to saturation point. Since mineral oil is the most widely used insulating liquid, the aging measurements were only carried out on this material. It has been discovered that using CeO_2_ nanoparticles slows the deterioration of mineral oil breakdown strength with age. The dielectric constant of mineral oil decreases with aging, while nano-oil sustains the aging stresses efficiently. The introduction of CeO_2_ NP with insulating oil is novel and shows the possibility of becoming a better future insulating fluid. Although many dielectric properties are studied in this work, many aspects are still left to be explored in future research.

## Author contribution statement

1 - Conceived and designed the experiments;

2 - Performed the experiments;

3 - Analyzed and interpreted the data;

4 - Contributed reagents, materials, analysis tools or data;

5 - Wrote the paper.

## Data availability statement

Data will be made available on request.

## Funding

This research is funded by Princess Nourah bint Abdulrahman University Researchers Supporting Project number (PNURSP2023R79), Princess Nourah bint Abdulrahman University, Riyadh, Saudi Arabia.

## Declaration of competing interest

The authors declare no conflict of interest.

The authors declare that they have no known competing financial interests or personal relationships that could have appeared to influence the work reported in this paper.
